# Presence of mechanical dyssynchrony in duchenne muscular dystrophy

**DOI:** 10.1186/1532-429X-13-12

**Published:** 2011-02-02

**Authors:** Kan N Hor, Janaka P Wansapura, Hussein R Al-Khalidi, William M Gottliebson, Michael D Taylor, Richard J Czosek, Sherif F Nagueh, Nandakishore Akula, Eugene S Chung, Woodrow D Benson, Wojciech Mazur

**Affiliations:** 1 The Heart Institute and Cincinnati Children's Hospital Medical Center, Cincinnati, Ohio, USA; 2 Department of Radiology, Cincinnati Children's Hospital Medical Center, Cincinnati, Ohio, USA; 3 Duke University School of Medicine, Durham, North Carolina, USA; 4 Methodist Hospital, Houston, Texas, USA; 5 The Heart and Vascular Center, The Christ Hospital, Cincinnati, Ohio, USA

## Abstract

**Background:**

Cardiac dysfunction in boys with Duchenne muscular dystrophy (DMD) is a leading cause of death. Cardiac resynchronization therapy (CRT) has been shown to dramatically decrease mortality in eligible adult population with congestive heart failure. We hypothesized that mechanical dyssynchrony is present in DMD patients and that cardiovascular magnetic resonance (CMR) may predict CRT efficacy.

**Methods:**

DMD patients (n = 236) were stratified into 4 groups based on age, diagnosis of DMD, left ventricular (LV) ejection fraction (EF), and presence of myocardial fibrosis defined as positive late gadolinum enhancement (LGE) compared to normal controls (n = 77). Dyssynchrony indices were calculated based on timing of CMR derived circumferential strain (e_cc_). The calculated indices included cross-correlation delay (XCD), uniformity of strain (US), regional vector of variance (RVV), time to maximum strain (TTMS) and standard deviation (SD) of TTMS. Abnormal XCD value was defined as > normal + 2SD. US, RVV, TTMS and SD were calculated for patients with abnormal XCD.

**Results:**

There was overall low prevalence of circumferential dyssynchrony in the entire DMD population; it increased to 17.1% for patients with abnormal EF and to 31.2% in the most advanced stage (abnormal EF with fibrosis). All but one DMD patient with mechanical dyssynchrony exhibited normal QRS duration suggesting absence of electrical dyssynchrony. The calculated US and RVV values (0.91 ± 0.09, 1.34 ± 0.48) indicate disperse rather than clustered dyssynchrony.

**Conclusion:**

Mechanical dyssynchrony is frequent in boys with end stage DMD-associated cardiac dysfunction. It is associated with normal QRS complex as well as extensive lateral fibrosis. Based on these findings, it is unlikely that this patient population will benefit from CRT.

## Background

In patients with New York Heart Association (NYHA) class III, ambulatory class IV systolic heart failure (HF) and recently class I and II, with electrocardiographic evidence of ventricular dyssynchrony, cardiac resynchronization therapy (CRT) improves quality of life and functional status, reduces heart failure-related hospitalizations, and prolongs survival [1-4]. While current indications are based on QRS duration > 120 ms, HF patients with narrow QRS complexes have been investigated for the presence of mechanical dyssynchrony. In this population, the prevalence of dyssynchrony is present though at a lower frequency compared to patients with prolonged QRS. Accordingly, in several small pilot studies [5-8] these patients appear to benefit from CRT, although a large multicenter randomized trial did not meet its primary endpoint [9]. In the pediatric heart failure population there is even less data and there are no large retrospective or prospective trials in reference to CRT [10].

A major cause of pediatric HF, Duchenne muscular dystrophy (DMD) is an X-linked recessive disorder characterized by disruption of the dystrophin protein. The resulting cell membrane degradation in skeletal and cardiac muscle tissue [11-13] leads to a cascade of inflammation, edema, necrosis and fatty replacement [14-17]. This ultimately results in the development of myocardial fibrosis. Although the extent of involvement varies, overt cardiac systolic dysfunction is present by 20 years of age in essentially all DMD patients. Current therapeutic options for DMD associated HF are limited with no established standard of care for medical or device interventions. This study sought to determine the feasibility of CRT in the DMD population, most of whom have normal QRS complexes. We evaluated the prevalence and character of mechanical dyssynchrony using the cross correlation delay (XCD) method, a highly objective, automatable dyssynchrony parameter on tagged cardiovascular magnetic resonance (CMR) [18].

## Methods

### Study Population

This is a retrospective cohort analysis and was conducted with the approval of the Institutional Review Board at the Cincinnati Children's Hospital Medical Center. Patients were identified using the clinical CMR database at the Heart Institute at Cincinnati Children's Hospital Medical Center from September 2005 to March 2009. This study included all DMD patients > 7 years of age who had previously undergone clinical CMR studies. Subjects < 7 years of age were excluded in order to eliminate the need for sedation to undergo the CMR study. A diagnosis of DMD was confirmed by a skeletal muscle biopsy and/or a DNA analysis demonstrating a characteristic dystrophin mutation.

Genetic testing was performed at Athena Diagnostics, Inc. or Medical Genetics Laboratories, Baylor College of Medicine (prior to 2006) and at Emory University Genetics Laboratory, Atlanta, Georgia (since 2006).

### Subject groups

**Figure 1:**
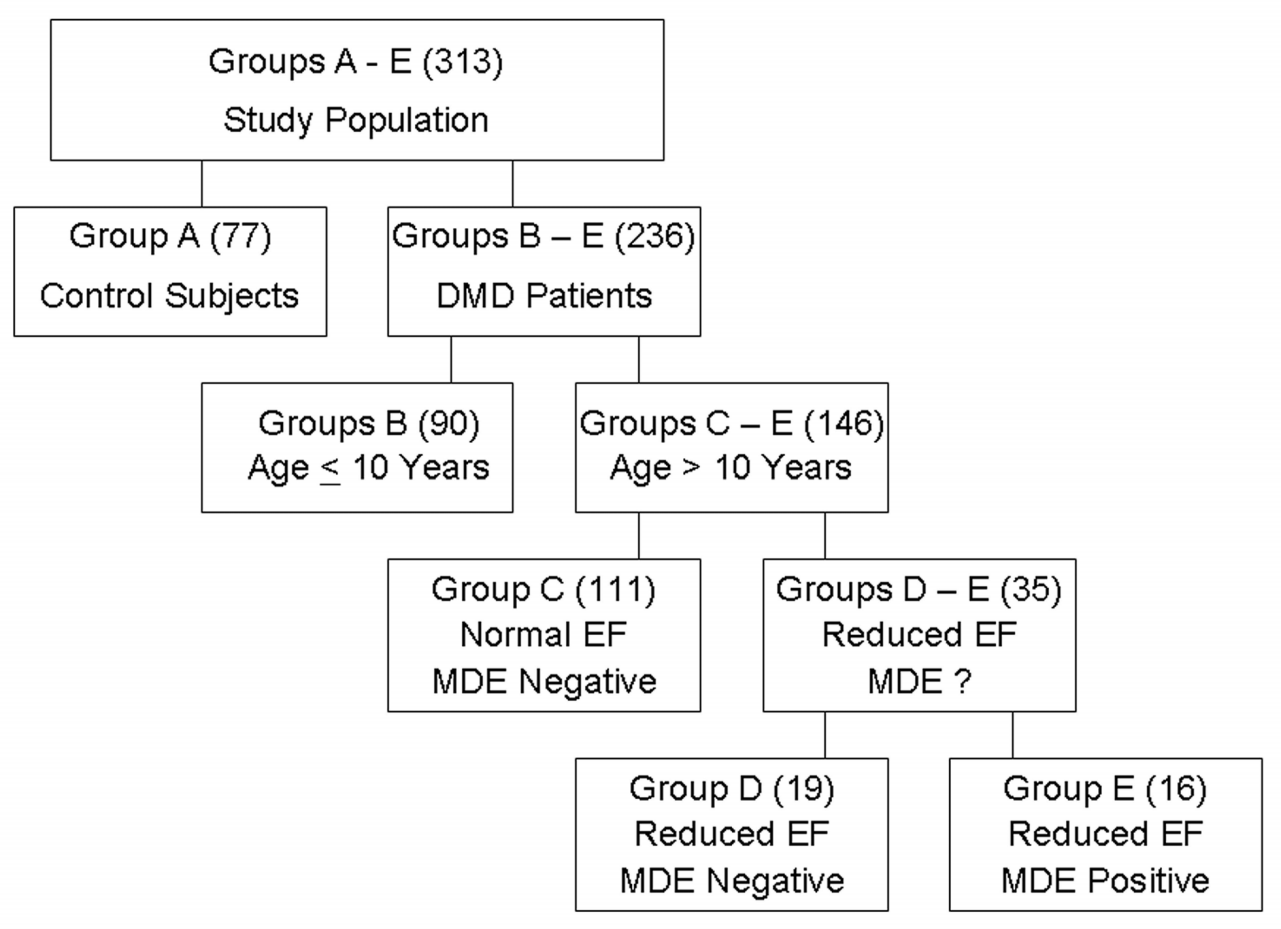
**Group Stratification**. DMD patients (n = 236) were stratified into 4 groups based on age, diagnosis of DMD, left ventricular (LV) ejection fraction (EF), and presence of myocardial fibrosis defined as positive late gadolinium enhancement(LGE). DMD and compared to normal controls.

DMD patients (n = 236) were stratified into 4 groups based on age, left ventricular (LV) ejection fraction (EF), presence of myocardial fibrosis defined as positive late gadolinum enhancement (LGE) and compared to normal controls (Figure 1).

### CMR and HARP^® ^Analyses

Clinical CMR studies were conducted on either a Siemens 3 Tesla *Trio *(Siemens Medical Solutions, Malvern, PA/Erlangen, Germany) or on a 1.5 Tesla GE Signa *Excite *(General Electric Healthcare; Milwaukee, WI). The type and field strength of MRI system was based solely on clinical availability and scheduled independent of the patient's clinical status or prior type or field strength of the first study.

Routine cardiac functional imaging was performed with a retrospectively vectorcardiographic (VCG) gated, segmented Steady State Free Precession (SSFP) technique after localized shimming and/or frequency adjusting. Subjects were breath-held as tolerated; for those subjects who could not adequately breath-hold, a free breathing technique with multiple signal averaging was used. Standard functional imaging included a short axis stack of cine SSFP images from cardiac base to apex; the short axis was prescribed as the perpendicular plane to the left ventricular long axis in 2 and 4 chamber views as per previously published protocols. Typical scan parameters were as follows: FOV = 32-38 cm, slice thickness = 5-6 mm, gap = 1-2 mm, NEX = 2 (breath hold; 4-5 for free breathing), TE/TR = 1.4/2.8 (Siemens), TE/TR = 2.0/4.0 (GE), in-plane resolution = 1.2- 2.2 mm. Minimums of 12 slices were performed with 20 phases/slice.

Tagged cine CMR images were acquired in the short axis of the mid ventricle at the level of the papillary muscles based on the four chamber view using an ECG-triggered segmented k-space fast gradient echo sequence with spatial modulation of magnetization in orthogonal planes. Grid tag spacing was 8 mm. The scan parameters used were: TE/TR = 3/15 ms. field of view = (30-32) × (25-26) cm, slice thickness = 6 mm, acquisition matrix = 256 × 144, flip = 20°, views per segments = 8-9. Only good quality tagged cine MRIs were included for analysis (confirmed by 2 independent expert readers: NA and KNH).

### Late gadolinum enhancement (LGE)

LGE imaging was performed on DMD patients when intravenous access was obtained (n = 121); no LGE imaging was performed in the control group. LGE imaging was performed via a FLASH inversion sequence recovery protocol 10 min after 0.2 mmol/kg gadolinium diethylenetriamine penta-acetic acid (Gd-DTPA) injection. LGE imaging was considered positive if any area of the mid-myocardium showed hyperenhancement as assessed by consensus of 3 readers (KNH, WMG, WM)

### ECG Analysis

Standard 12 lead ECG was obtained in all patients; QRS duration was measured manually.

### Data Analysis

#### Left ventricular function and volumes

Left ventricular volumes, mass and global function were assessed via standard planimetry techniques using semi automated computer software (QMASS v.6.1.5, Medis Medical Imaging Systems, Netherlands) by expert readers (KNH and WMG). Left ventricular stroke volume was also determined by analysis of cine velocity encoded phase contrast gradient echo (GRE) sequence of the ascending aorta via Medis QFLOW^® ^platforms for internal validation of the stroke volume determined by ventricular planimetry. Ventricular volumes, mass, and ejection fraction were tabulated for each subject.

#### Myocardial Strain Analysis

As previously described, tagged images from two times points of the mid-ventricle were analyzed using the HARmonic Phase (HARP, Diagnosoft, CA, USA) technique [19-23]. Details of circumferential strain (ε_cc_) analysis have been previously described [24,25]. Only the mid-ventricular slice was analyzed secondary to [24,25] the limited reproducibility of the analysis of the basal and apical slices. The regional (absolute value and time to peak) and global ε_cc _data was exported to a spreadsheet file for analysis.

#### Late Gadolinum Enhancement Analysis

LGE transmural extent was calculated as the ratio of myocardial LGE thickness to total wall thickness. It was expressed in 2 categories: <50%, and >50%.

#### Dyssynchrony Assessment

ε_cc _vs. time curves from 24 sub-regions at the mid ventricular level were used. Six regional ε_cc _curves were constructed by taking the average of four adjacent sub-regions to represent the segments 7-12 in the AHA 17 segment heart model. Sub-regional ε_cc _curves were used in the analysis of spatial dyssynchrony quantified by Uniformity of Strain (US), and Regional Vector of Variance (RVV). Regional ε_cc _curves were used in the analysis of temporal dyssynchrony quantified by cross-correlation delay (XCD), time to maximum strain (TTMS) and the standard deviation of TTMS (STD). Data were analyzed using custom developed software using IDL (IDL 6.2, ITT Visual Information Solutions, Boulder, CO).

(1) The cross-correlation delay (XCD)(18) between two curves was computed by shifting one curve in time relative to the other curve and computing the normalized correlation between the curves for each time shift. The time shift between the two curves that resulted in the maximum correlation value was deӿned as the temporal delay between the two curves. A temporal delay was computed between the anterior and inferior segments (XCD 7-10), the anteroseptal and inferolateral segments, (XCD 8-11) and the inferoseptal and anterolateral segments, (XCD 9-12) and the reported XCD was the maximum of these three delays. As normal values for above variables have not been published, we used values ≥ mean + 2 standard deviations for XCD as indicative for dyssynchrony.

(2) Time to maximum strain (TTMS) was calculated as follows: for each regional ε_cc _curve, the time from the onset of QRS to the maximum absolute ε_cc _(T_max_) was determined. Then TTMS was calculated as the difference between the shortest and the longest T_max _i.e. the time difference between the earliest and the latest time at which a region reaches its maximum strain.

(3) The standard deviation (STD) of T_max _is calculated for each of the six regions.

**Figure 2:**
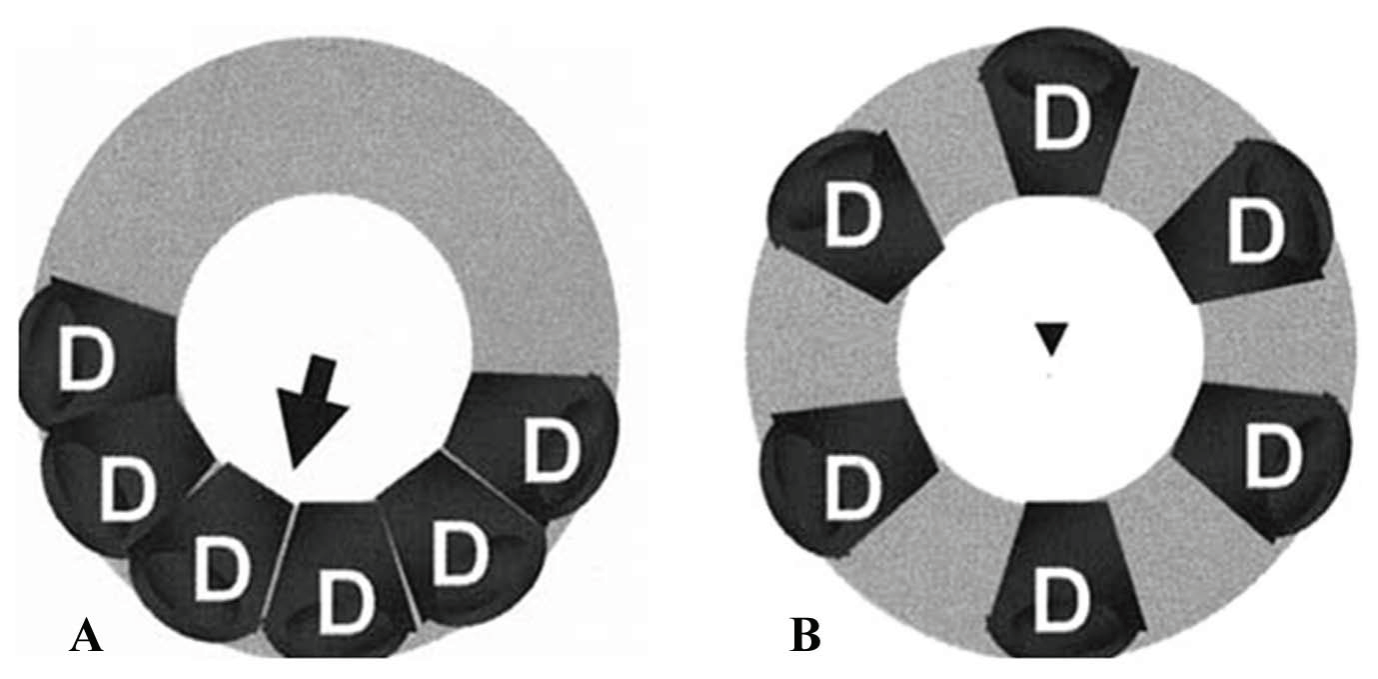
**Schematic representation of clustered (A) and disperse (B) dyssynchrony**. Depending how dyssynchrony is being indexed, Results could be the same for both situations, despite markedly different impact on dsyynchrony. In example B, there is a "net" synchronous contraction. Uniformity of Strain (US) and Regional Variance of Strain (RVV) are vector derived indices differentiating clustered from dispersed dyssynchrony. Adapted from: Lardo, A. C. et al. J Am Coll Cardiol 2005;46:2223-2228.

In addition to the above, we evaluated two additional indices of "global dyssynchrony" differentiating between "clustered" and "dispersed "dyssynchrony (Figure 2) [26].

(1) Uniformity of Strain (US) [27] was derived by subjecting the spatial plot of sub-regional strains at end systole (defined by the time at which mean ε_cc _reached maximum) to Fourier analysis. If all sub-regions shorten simultaneously (synchronously), the plot appears as a straight line, with power only in the zero-order Fourier term (A_0_), whereas regionally clustered dyssynchrony generates an undulating plot with higher power in the first-order term (A_1_). The US index is defined as $\sqrt{A_{0}^{2}/\left(A_{0}^{2}+2A_{1}^{2}\right)}$. The US index is zero for dyssynchronous contraction and 1 for perfectly synchronous contraction.

(2) Regional vector of variance (RVV) [28] was derived by representing the ε_cc _value of the n^th ^sub-region at a given time point by a vector with magnitude ε_cc _(n) and phase 2πn/24, (n = 1..24). RVV was calculated as the vector sum of all ε_cc _(n) giving a zero value for perfect synchrony. The maximum value of RVV depends on the number of segments analyzed. For 24 segments the maximum RVV value is 7.66.

Patients with abnormal XCD were presumed to have mechanical dyssynchrony and TTMS, SD, RVV, US were also calculated and compared for groups A-E.

#### Statistical Analysis

Results are expressed as mean ± SD for continuous data and as percentages and numbers for categorical data. Pearson's correlation coefficients were calculated to assess for linear relationships between relevant variables. Dunnett test was used to compare groups (B-E) to the control group (A) on variables of interest.

## Results

236 consecutive patients with DMD where evaluated. Patient demographic, EF, mass volume ratio and mean values for all dyssynchrony indices are presented in Table 1. All the normal controls had absolute ε_cc _greater than 17 and all DMD patients had absolute ε_cc _less than 17. In DMD patients but not normal controls, strain declined with age as previously described [24].

**Table 1:** General Characteristic by Group

Parameter	Group				
	
	A (n = 77)	B (n = 90)	C (n = 111)	D (n = 19)	E (n = 16)
Age	13.9 ± 8.9	8.5 ± 0.9**	12.9 ± 2.8	15.0 ± 3.9	17.3 ± 5.4**

LVEF (%)	64.6 ± 5.9	65.0 ± 4.8	64.4 ± 5.8	49.4 ± 6.6**	36.5 ± 12.2**

XCD^a^	45.6 ± 16.9	35.3 ± 16.6	38.2 ± 19.8	52.2 ± 22.4	73.0 ± 41.9

US_max	0.97 ± 0.04	0.98 ± 0.03	0.97 ± 0.03	0.97 ± 0.04	0.92 ± 0.08**

TTMS	78.2 ± 31.8	65.4 ± 22.9**	71.2 ± 24.0	95.1 ± 19.6**	111.4 ± 33.0**

RVV_max	0.95 ± 0.57	1.03 ± 0.55	1.13 ± 0.57	1.03 ± 0.51	1.34 ± 0.57**

STD_peak	31.7 ± 12.0	26.7 ± 8.4**	29.6 ± 9.7	36.5 ± 6.9	46.1 ± 16.6**

QRS	92.8 ± 11.4^1^	85.1 ± 7.8**	86.6 ± 8.0**	86.1 ± 13.4	97.1 ± 12.9

ε_cc_	-18.2 ± 4.5	-14.2 ± 1.4**	-13.2 ± 2.0**	-10.7 ± 2.1	-7.0 ± 2.8

**Figure 3:**
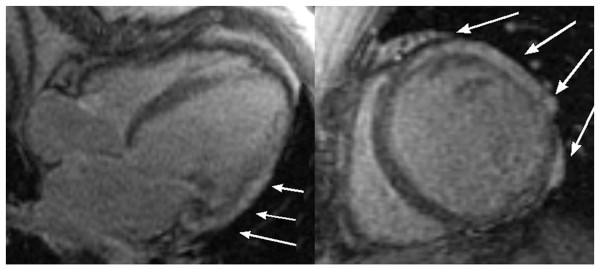
**Late gadolinum enhancement (LGE)**. LGE images of a representative patient from group E. Note predominantly transmural enhancement involving anterolateral and inferolateral segments in apical four chambers and short axis views.

LGE was observed in group E only, involving anterolateral and inferolateral segments, with extent of enhancement over 50% of wall thickness (Figure 3).

### QRS duration

Summary statistics for QRS duration by groups are given in Table 1. Neither left nor right bundle branch block morphology was observed in our study group. There were 3 boys (1 in each group A, D and E) with QRS duration over 120 ms consistent with nonspecific intraventricular conduction delay.

### Dyssynchrony

Comparison of boys with identified dyssynchrony to control group is presented in Table 2. We defined XCD to be the largest of the three XCD (XCD 7-10, XCD 8-11 or XCD 9-12) measurements. There were 9 (3.8%) boys (3 in group C, 1 in group D and 5 in group E) with abnormal XCD, defined as XCD larger than 79.4 ms (calculated as mean + 2SD of XCD of normal control) In these 9 patients, TTMS was 111.2 ± 39.5 ms, RVV was 1.3 ± 0.5, STD was 45.6 ± 18.1, US was 0.9 ± 0.10 and QRS duration was 98.2 ± 15.3 ms. Only 1 of those 9 boys had a QRS > 120 ms. One-way ANOVA model comparing dyssynchrony indices in 9 DMD boys with mechanical dyssynchrony to those of control group A (n = 77) showed statistically significant differences in all indices except for QRS duration (p = 0.2825). In addition, RVV index was borderline significant (p = 0.0538) as compared to the control group. It needs to be noted that despite statistical differences US and RVV values in boys with dyssynchrony are suggestive of nearly "net" synchronous contraction (disperse rather than clustered dyssynchrony) (Figure 2).

**Table 2:** Abnormal XCDs vs. the control

Parameter	Group	
	
	Control (n = 77)	Abnormal XCDs (n = 9)
Age	13.9 ± 8.9	16.7 ± 5.0

LVEF (%) (min, max)	64.6 ± 5.9	43.9 ± 16.7**

LVRI	0.71 ± 0.19	0.66 ± 0.21

XCD^a^	45.6 ± 16.9	116.5 ± 20.5**

Presence of LGE	0 (0.0%)	4 (57.1%)**

US_max	0.97 ± 0.04	0.91 ± 0.09**

TTMS	78.2 ± 31.8	111.2 ± 39.5**

RVV_max	0.95 ± 0.57	1.34 ± 0.48

STD_peak	31.7 ± 12.0	47.6 ± 18.1**

QRS	92.8 ± 11.4^1^	98.2 ± 15.3

**P < 0.05 as compared to the control group (A) using Dunnett test.

^a^XCD = maximum of (XCD 1-4, XCD 2-5 and XCD 3-6).

## Discussion

This study demonstrated that there is a significant prevalence of mechanical ventricular dyssynchrony (17%) in DMD patients with EF < 55%. Additionally, the prevalence of dyssynchrony was greater in patient subsets with more advanced cardiac disease (low EF and fibrosis).

This is the first study to our knowledge to use an automated MRI derived cross correlation technique for assessment of mechanical dyssynchrony. It is also the first study to assess prevalence and characteristics of dyssynchrony in a large DMD patient population. Previous application of the XCD method utilized echocardiography [29] data. Subsequently, CMR (phase contrast) [30,31] was found to be superior for measuring time to peak dyssynchrony parameters for discriminating between subjects with and without dyssynchrony. Circumferential strain is an established CMR method of dyssynchrony assessment, Han et al evaluated circumferential myocardial strain in cardiomyopathy with and without left bundle branch block; these authors assessed opposing wall delays, and reported values within the range of XCD values in our patient population [32]. The current study, to our knowledge is also the first to assess circumferential dyssynchrony by XCD in a narrow QRS patient population.

Boys with DMD do not fulfil classic adult derived criteria for CRT: QRS complex is narrow in 97% of the population, EF is generally greater than 35%, and due to the nature of the disease, NYHA functional class could not be assessed. These barriers, combined with compelling and devastating disease progression, make it even more important to develop sensitive tools to create prediction models as to likelihood of success with CRT in all or a distinct subset of this patient population. In this study we demonstrated a low prevalence of dyssynchrony in the overall population of boys with DMD using cross-correlation delay; but the prevalence increased to 17.1% for patients with abnormal EF (groups D and E) and to 31.2% in the most advanced stage (group E). All but one of the boys with DMD and mechanical dyssynchrony exhibited normal QRS duration suggesting absence of electrical dyssynchrony. Only weak relationships were found between dyssynchrony indices and ε_cc _or EF likely due to low number of boys with low EF. Of interest, in those with dyssynchrony, US and RVV values indicate the presence of disperse rather than clustered dyssynchrony. Disperse dyssynchrony patients would not be expected to respond favourably to CRT (Figure 2).

Location of scar tissue is concerning as well, as most common LGE location was in the inferolateral segments. In patients with ischemic CMP, inferolateral scar involving over 50% of wall thickness virtually excluded improvement with CRT [33], as did total scar volume of over 15% [34,35]. We could not assess total scar volume in our study as only 3 short axis slices were acquired for LGE assessment. Even if left ventricular lead was implanted before the development of extensive inferolateral fibrosis, it is possible that disease progression in that segment would diminish efficacy of CRT over time.

Do these barriers preclude the possibility that LV pacing might benefit these patients? The favourable effect of LV pacing on the failing heart's contractility and external constraint may help to slow the progression of disease. LV pacing also reduces the work performed by the LV as well as beat to beat transmural stress. It is possible that with chronic treatment, a small change in detrimental physiology might realize clinical benefit. Further studies are warranted to evaluate these hypotheses.

## Study Limitations

We had a relatively small number of patients with low EF and mechanical dyssynchrony; all had advanced DMD associated cardiac disease (2 from group D and 7 from group E). All of the patients in group E had extensive fibrosis, obviously due to selection bias less advanced cases were missed. To prove that our analysis is valid, response to biventricular pacing would be most optimal. Tag analysis was performed up to 3rd frame after peak systole, (due to tag fading), it is possible that very dyssynchronous segments were missed, however the prevalence of mechanical dyssynchrony in boys with most advanced disease was similar to that seen in adult heart failure patients.

## Conclusions

Mechanical dyssynchrony was seen in 17.1% of DMD boys with EF < 55% and in 31.2% of boys with most advanced stages of disease (low EF and fibrosis); however, the observed dyssynchrony was dispersed in nature. It remains to be seen if those patients would respond to CRT.

## Competing interests

The authors declare that they have no competing interests.

## Authors' contributions

KNH, WM and WMG contributed to all aspects of the manuscript's conception, design, data analysis, collection, critical revision and final approval. JPW, MDT, HAR, SFN, NA, ESC and DWB contributed in data analysis, critical revision and final approval of the manuscript.
